# Prevalence of Hypertension and Associated Factors in a City in Madagascar

**DOI:** 10.7759/cureus.76735

**Published:** 2025-01-01

**Authors:** Rova Malala Fandresena Randrianarisoa, Sakaiza Malala Florine Randrianambininjanahary, Narindrarimanana Avisoa Randriamihangy, Fidiarivony Ralison

**Affiliations:** 1 Internal Medicine, University Hospital Joseph Raseta Befelatanana, Antananarivo, MDG; 2 Department of Medical Imaging, Joseph Ravoahangy Andrianavalona Hospital, Antananarivo, MDG; 3 Department of Cardiology, Mahavoky Atsimo University Hospital, Mahajanga, MDG; 4 Department of Internal Medicine, Mahavoky Atsimo University Hospital, Mahajanga, MDG

**Keywords:** cardiovascular risk factors, hypertension, madagascar, prevalence, public health

## Abstract

Introduction

In recent decades, the prevalence of hypertension has decreased in high-income countries but remains high in sub-Saharan Africa. However, epidemiologic data are still lacking in some regions, such as Madagascar. This study shows the reality of hypertension and cardiovascular health in Africa's remotest regions. Our aim was to report the prevalence of hypertension and its associated factors in a city in Madagascar.

Materials and methods

This was a cross-sectional descriptive and analytical study conducted in 2019, including adults aged ≥ 18 years residing in the city of Toliara, located in the south of Madagascar. Hypertension was defined according to the recommendations of the European Society of Cardiology and the European Society of Hypertension.

Results

Of the 331 individuals included in the study, 62 (18.73%) had hypertension. The prevalence was 22.70% in men (n = 37) and 14.88% in women (n = 25), and 61.54% in those aged ≥ 60 years. Grade 1 hypertension was found in 45.16% (n = 28) and both systolic and diastolic hypertension in 43.55% (n = 27). The rates of treated and controlled hypertension were 45.16% (n = 28) and 17.74% (n = 11), respectively. After statistical adjustment, advanced age (p = 0.001) and short sleep duration (p = 0.004) were significant associated factors.

Conclusions

The prevalence of hypertension was low. The associated factors were well described in the literature. The rates of treated and controlled hypertension were very low, reflecting a failure of our healthcare system.

## Introduction

Hypertension is the most common modifiable cardiovascular risk factor worldwide. It is a serious chronic disease and one of the leading causes of death from cardiovascular disease (CVD) [1]. Hypertension increases the risk of cardiovascular events such as chronic renal failure, stroke, myocardial infarction and heart failure [2]. Modifiable risk factors include unhealthy diet, physical inactivity, tobacco and alcohol use, overweight or obesity, and diabetes. Non-modifiable risk factors include family history of hypertension, gender, advanced age, and race [3].

According to the WHO, the number of adults with hypertension worldwide has doubled from 650 million in 1990 to 1.3 billion in 2019 [4]. Hypertension is estimated to affect 33% of adults aged 30-79 years. In high-income countries and European regions, the prevalence has decreased in recent decades. In contrast, slight increases have been observed in the Western Pacific and Southeast Asian regions [4]. In sub-Saharan Africa, the prevalence of hypertension remains higher and the mortality rate is three times higher, with 22.9 million deaths attributed to CVD annually [5]. The WHO had set a target of a 25% relative reduction in the prevalence of hypertension in sub-Saharan Africa by 2025 [6].

In Madagascar, approximately 61% of CVD deaths are due to high systolic blood pressure (SBP). The prevalence of age-standardized hypertension among adults aged 30-79 years is 37%, according to the WHO report for 2023 [7]. Hypertension is therefore a major public health problem. However, epidemiological data on hypertension are still lacking in certain regions of the country, especially in Toliara. Conducting this study will allow us to establish the cardiovascular health profile of this specific population and add new information on the distribution of risk factors in the African literature.

Our objective was to report the prevalence of hypertension and its associated factors in adults in the urban area of the city of Toliara.

## Materials and methods

Study characteristics and population

**Figure 1:**
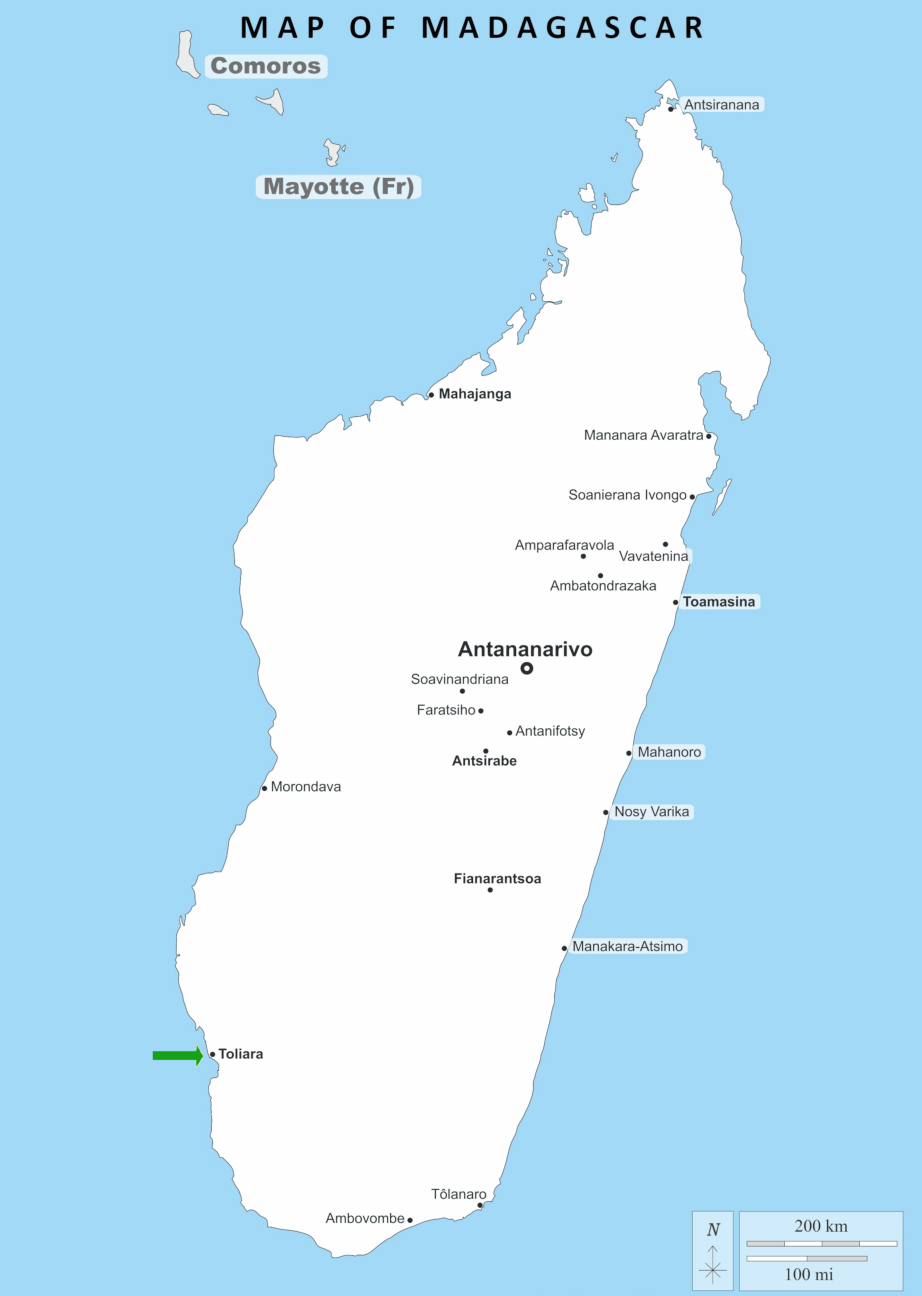
Map of Madagascar Green arrow: location of the city of Toliara Image source: https://d-maps.com

This was a descriptive and analytical cross-sectional study conducted over a two-month period from January to March 2019. Residents of the urban area of Toliara were included in the study. Toliara is a city in the south of Madagascar (Figure 1). In recent years, there has been a spectacular demographic explosion due to migration. The population consists of an indigenous ethnic group, the Vezo, mixed with migrant groups [8].

Sampling

According to the 2019 census, the urban area included 170,194 residents in seven arrondissements and 42 neighborhoods [9]. Fourteen neighborhoods were randomly selected (two neighborhoods in each arrondissement). With a confidence level of 95% and a margin of error of 6%, the sample size was 266 (a minimum of 19 people per neighborhood). Individuals were randomly selected by going door-to-door in each neighborhood.

Inclusion and exclusion criteria

We included men and women over 18 years of age who volunteered to participate after being informed of the study objectives and procedures. We excluded pregnant women and participants who decided to withdraw during the study.

Study variables

We collected sociodemographic characteristics, cardiovascular risk factors, and sleep duration. Height and waist circumference were measured using a tape measure, and weight was measured using an electronic scale. Body mass index (BMI) was calculated from weight and height. Nutritional status was classified according to BMI: underweight, if BMI < 18 kg/m^2^; healthy weight, if BMI between 18-25 kg/m^2^; overweight, if BMI between 25-30 kg/m^2^; obesity, if BMI ≥ 30 kg/m^2^. For abdominal obesity, we used Asian criteria, which define it as a waist circumference > 90 cm for men and > 80 cm for women [10]. This choice is explained by the lack of a national consensus for abdominal obesity and the greater physical similarity of the Malagasy population to the Asian population.

Physical activity was defined according to the weekly frequency of travel-related activities (walking, cycling, commuting to work, shopping or leisure activities). People were considered active if they engaged in these activities for at least 30 minutes per day, five days per week. With regard to smoking, we considered anyone who used or had used tobacco to be a smoker.

Blood pressure measurement and definition of hypertension

For each participant, blood pressure measurements were taken in the sitting position at the second, fifth, and 10th minute of rest. For the first measurement, both arms were used and the highest value was selected. The arm with the highest values was then used for subsequent measurements. The values of the last two measurements were averaged to obtain the measured blood pressure. A Spengler electronic sphygmomanometer, validated by the European Society of Cardiology, was used throughout the study.

We used the 2018 European Society of Cardiology and European Society of Hypertension (ESC/ESH) definition [11]. Any person with a mean SBP ≥ 140 mmHg and/or diastolic blood pressure (DBP) ≥ 90 mmHg was considered hypertensive. Anyone on antihypertensive treatment was considered hypertensive. Hypertension was classified and graded according to severity: grade 1 for SBP 140-159 and/or DBP 90-99 mmHg, grade 2 for SBP 160-179 and/or DBP 100-109 mmHg, and grade 3 for SBP ≥ 180 and/or DBP ≥ 110 mmHg. Isolated systolic hypertension was defined as SBP ≥ 140 and DBP < 90 mmHg, and isolated diastolic hypertension as SBP < 140 and DBP ≥ 90 mmHg [11]. Controlled hypertension was defined as SBP < 140 mmHg and DBP < 90 mmHg and taking medication for hypertension. Familial hypertension was considered if a first- or second-degree relative had been diagnosed with hypertension by a physician.

Statistical analysis

Data were entered and analyzed using Epi Info® software, version 7 (Centers for Disease Control and Prevention, Atlanta, GA, USA). Quantitative variables were expressed as means. Qualitative variables were presented as frequencies and percentages. Chi-squared test and Fisher’s exact test were used for univariate analysis. "Don’t know" responses for some variables were not analyzed. P-values < 0.05 were considered statistically significant. The explanatory variables associated with p-value < 0.05 were then analyzed by logistic regression. The multivariate logistic regression model was used with stepwise backward elimination of nonsignificant variables, with hypertension status as the dependent variable. P-values < 0.05 were considered statistically significant.

Ethical considerations

The study was approved by the Research Ethics Committee of the University of Mahajanga (approval UMG-2019) and validated by the Atsimo Andrefana Regional Public Health Department. Each participant was informed about the objectives of the study, the use of the data for research purposes, and the anonymity of the data collected.

## Results

A total of 331 individuals were included. The sex ratio was 0.97, with 163 men and 168 women. The mean age was 37.92 years (±13.92), with extremes of 18 and 85 years. The mean BMI was 23.66 kg/m^2^ (±5.22). Table 1 shows the sociodemographic characteristics of the population. Table 2 shows cardiovascular risk factors. Table 3 shows the mean blood pressure values of the general population and the hypertensive individuals.

**Table 1:** Sociodemographic characteristics of the population

Sociodemographic characteristics		Number (N = 331)	Percentage (%)
Gender	Women	168	50.75
	Men	163	49.24
Age group (years)	18-40	194	58.61
	40-60	111	33.53
	≥60	26	7.85
Family situation	Living with partner	196	59.21
	Living with parents	54	16.31
	Living with children	50	15.11
	Living alone	31	9.36
Family role	Mother of the family	133	40.18
	Father of a family	114	34.44
	Adult without child	23	6.95
	Child status	61	18.43
Educational level	Illiterate	11	3.32
	Primary	45	13.59
	Middle school	99	29.91
	High school	97	29.30
	University	79	23.87

**Table 2:** Cardiovascular risk factors of the population

Cardiovascular risk factors		Number (N = 331)	Percentage (%)
Diabetes	Yes	6	1.81
	No	47	14.20
	Don’t know	278	83.99
Familial hypertension	Yes	192	58
	No	82	24.77
	Don’t know	57	17.22
Body mass index	Underweight	41	12.39
	Healthy weight	183	55.29
	Overweight	74	22.36
	Obesity	33	9.97
Abdominal obesity	Yes	117	35.35
	No	214	64.65
Smoking	Yes	112	33.84
	No	219	66.16
Physical activity	Active	79	23.87
	Inactive	252	76.13
Sleep duration (hours)	≤6	117	35.36
	7-9	171	51.66
	≥9	43	12.99

**Table 3:** Means values of arterial blood pressure SBP: systolic blood pressure; DBP: diastolic blood pressure

Blood pressure	General population			Hypertensive individuals		
	Men (n = 163)	Women (n = 168)	Total (N = 331)	Men (n = 37)	Women (n = 25)	Total (n = 62)
SBP (mmHg)	124.28 (±19.51)	115.32 (±19.01)	119.73 (±19.75)	151.32 (±17.03)	144.64 (±24.83)	148.63 (±20.61)
DBP (mmHg)	75.11 (±11.65)	72.75 (±11.81)	73.91 (±11.77)	89.38 (±11.56)	90.84 (±13.02)	89.97 (±12.09)

**Figure 2:**
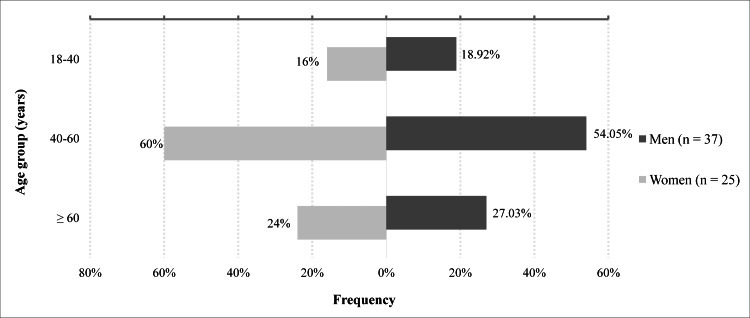
Prevalence of hypertension by gender and age group

Sixty-two individuals were hypertensive, representing a prevalence of 18.73%. Their mean age was 51.40 years (±12.88). The prevalence of hypertension was 22.70% in men (n = 37) and 14.88% in women (n = 25). The prevalence was 61.54% among those ≥ 60 years of age. Figure 2 shows the frequency of hypertension by gender and age group.

Grade 1 hypertension was found in 45.16% (n = 28), grade 2 in 22.58% (n = 14) and grade 3 in 6.45% (n = 4) of cases. Hypertension was both systolic and diastolic in 43.55% (n = 27), isolated systolic in 30.64% (n = 19) and diastolic in 8.06% (n = 5) of cases. Of the 62 hypertensive individuals, 28 (45.16%) were taking antihypertensive drugs and 11 (17.74%) had their blood pressure under control.

In univariate analysis, the factors significantly associated were: age groups 40-60 years (crude odds ratio (cOR) = 7.66, p = 0.001) and ≥ 60 years (cOR = 26.61, p = 0.001), family situation "living with children" (cOR = 2.06, p = 0.027), role "mother of the family" (cOR = 6.82, p = 0.001) and "father of the family" (cOR = 12.53, p = 0.001), overweight (cOR = 3.03, p = 0.001), obesity (cOR = 3.61, p = 0.003), abdominal obesity (cOR = 3.80, p = 0.001), and sleep duration of six or less hours (cOR = 2.74, p = 0.001) (Table 4).

**Table 4:** Factors associated with hypertension aOR: adjusted odds ratio; cOR: crude odds ratio; CI: confidence interval; ND: not defined; Ref: reference value

Sociodemographic characteristics and comorbidities		Hypertension		Total (N = 331)	cOR (CI_95%_)	p-value	aOR (CI_95%_)	p-value
		Yes n (%)	No n (%)					
Gender	Women	25 (14.88)	143 (85.12)	168	Ref			
	Men	37 (22.70)	126 (77.30)	163	1.67 (0.95-2.94)	0.046	6.65 (0.63-69.84)	0.113
Age group (years)	18-40	11 (5.67)	183 (94.33)	194	Ref			
	40-60	35 (31.53)	76 (68.47)	111	7.66 (3.69-15.87)	0.001	3.79 (1.66-8.65)	0.001
	≥60	16 (61.54)	10 (38.46)	26	26.61 (9.81-72.15)	0.001	17.42 (5.49-55.24)	0.001
Family situation	Living with partner	42 (21.43)	154 (78.57)	196	Ref			
	Living with parents	2 (3.70)	52 (96.30)	54	0.14 (0.03-0.60)	0.001	1.12 (0.06-19.10)	0.935
	Living with children	18 (36)	32 (64)	50	2.06 (1.05-4.03)	0.027	1.64 (0.71-3.80)	0.241
	Living alone	0	31 (100)	31	ND	0.001	ND	0.961
Family role	Mother of the family	25 (18.80)	108 (81.20)	133	6.82 (1.56-29.84)	0.001	2.61 (0.13-51.61)	0.526
	Father of the family	34 (29.82)	80 (70.18)	114	12.53 (2.89-54.20)	0.001	0.91 (0.06-12.43)	0.946
	Adult without child	1 (4.35)	22 (95.65)	23	1.34 (0.11-15.53)	0.62		
	Child status	2 (3.28)	59 (96.72)	61	Ref			
Educational level	Illiterate	0	11 (100)	11	ND	0.737		
	Primary	8 (17.78)	37 (82.22)	45	0.59 (0.23-1.48)	0.186		
	Middle school	16 (16.16)	83 (83.84)	99	0.53 (0.25-1.10)	0.065		
	High school	17 (17.53)	80 (82.47)	97	0.58 (0.28-1.20)	0.102		
	University	21 (26.58)	58 (73.42)	79	Ref			
Diabetes	Yes	5 (83.33)	1 (16.67)	6	6.19 (0.67-57.15)	0.087		
	No	21 (44.68)	26 (55.32)	47	Ref			
Familial hypertension	Yes	43 (22.40)	149 (77.60)	192	1.53 (0.77-3.03)	0.142		
	No	13 (15.85)	69 (84.15)	82	Ref			
Body mass index	Underweight	1 (2.44)	40 (97.56)	41	0.15 (0.02-1.20)	0.028	0.12 (0.01-1.22)	0.074
	Overweight	24 (32.43)	50 (67.57)	74	3.03 (1.59-5.77)	0.001	1.53 (0.59-3.98)	0.377
	Obesity	12 (36.36)	21 (63.64)	33	3.61 (1.58-8.24)	0.003	2.71 (0.77-9.49)	0,117
	Healthy weight	25 (13.66)	158 (86.34)	183	Ref			
Abdominal obesity	Yes	38 (32.48)	79 (67.52)	117	3.80 (2.14-6.76)	0.001	2.77 (0.97-7.90)	0.055
	No	24 (11.21)	190 (88.78)	214	Ref			
Smoking	Yes	24 (21.43)	88 (78.57)	112	1.29 (0.73-2.29)	0.225		
	No	38 (17.35)	181 (82.65)	219	Ref			
Physical activity	Inactive	49 (19.44)	203 (80.56)	252	1.22 (0.62-2.39)	0.339		
	Active	13 (16.46)	66 (83.54)	79	Ref			
Sleep duration (hours)	≤6	35 (29.91)	82 (70.09)	117	2.74 (1.52-4.96)	0.001	2.76 (1.35-5.66)	0.004
	7-9	23 (13.45)	148 (86.55)	171	Ref			
	≥9	4 (9.30)	39 (90.70)	43	0.66 (0.21-2.02)	0.328		

In multivariate analysis, significant factors were: age groups 40-60 years (adjusted odds ratio (aOR) = 3.79, p = 0.001) and ≥ 60 years (aOR = 17.42, p = 0.001) and sleep duration of six or less hours (aOR = 2.76, p = 0.004) (Table 4).

## Discussion

The distribution of CVD varies by socioeconomic status, race, and genetic factors [12]. Knowledge of cultural differences is essential to improve outcomes in the management of CVD. Observational evidence suggests that the risk of cardiovascular events is higher in Black adults than in non-Black adults [5]. In the present study, we examined the Malagasy population, a Black population with limited epidemiologic data on cardiovascular health in the general literature.

Overall prevalence of hypertension

The prevalence of hypertension found in this study was 18.73% among adults aged 18 to 85 years living in the city of Toliara. A few studies on the prevalence of hypertension have been reported in the Malagasy literature. In Antananarivo, the capital of Madagascar, the prevalence was 28.05% in 2009 [13]. In Antsirabe, the third largest city in the central highlands, the prevalence was 22.18% in 2005 [14]. In Moramanga, a commune located between the capital and the east coast of the country, the prevalence was 29.7% in urban areas and 27% in rural areas in 2015 [15]. In Mahajanga, a city in the northwestern coastal region of Madagascar, the prevalence was 23.3% in 2018 [16]. In Mandena, an agricultural village in the northern part of the east coast, the prevalence was 49.1% in 2018 [17]. Our prevalence is therefore lower than the results observed in these regions. The discrepancy between these studies may be explained by the geographical distribution of hypertension, which is influenced by socio-cultural inequalities and environmental factors [12]. Differences in methodology, such as the diagnosis of hypertension, may influence the prevalence estimates.

Within Africa, results on the prevalence of hypertension remain highly variable. Analyzing data from the African literature, Adeloye et al. reported higher frequencies in South Africa (77.3%), Tanzania (69.9%), Tunisia (69.3%), and Senegal (65.4%), and lower frequencies in Sudan (7.5%) and Ethiopia (9.9%) [18]. However, sub-Saharan Africa has the highest prevalence of hypertension in the world. Infectious and noncommunicable diseases coexist, and CVD is an increasing contributor to the global burden of disease [5].

Prevalence of hypertension by gender and age

According to gender, the prevalence of hypertension in our study was higher in men (22.70% vs. 14.88% in women). Blood pressure is a sexually dimorphic trait, and prevalence can vary significantly between men and women throughout life [19]. In the 2021 U.S. Heart Disease and Stroke Statistics update, the age-adjusted prevalence of hypertension in people over 20 years of age was higher in men (51.7% vs. 42.8%) [20]. Data from the CONSTANCES cohort of 59,805 individuals in France showed similar results [21]. Sex differences result from a combination of biological and psychosocial factors [19].

According to age, our prevalence was highest (61.54%) in people over 60 years. According to the ESC/ESH, the prevalence is estimated to be around 60% in people in their 60s and 70s [11]. The higher prevalence of hypertension in the elderly population can be explained by the natural age-related hardening of the arteries and aortas. Ageing is associated with changes in the vascular wall and increased arterial stiffness [22]. These older people are more likely to develop cardiovascular complications.

Treated and controlled hypertension

Forty-five percent (45.16%) of hypertensive individuals were treated, and the proportion with controlled hypertension was 17.74%. In the WHO 2023 report [4], treatment coverage is higher in the Americas (60%) and lower in the African regions (27%). Rates of controlled hypertension are higher in high-income countries (36%) and lower in low-income countries (11%) and African regions (12%). As the prevalence of hypertension increases, these low rates of treatment and control of hypertension will place an increasing burden of CVD on African regions. Our findings may be explained by the lack of availability and access to treatment and the absence of specific recommendations for the management of hypertension in Black adults living in Africa. Most international recommendations are based on data from high-income countries [5].

Factors associated with hypertension

The prevalence of hypertension was significantly associated with increasing age, and this association remained significant after statistical adjustment. Similar results have been observed in the Malagasy literature [15-17]. Advanced age is a non-modifiable cardiovascular risk factor and several mechanisms explain its role in hypertension: reduced compliance of proximal arteries, increased peripheral vascular resistance, reduced sensitivity of baroreceptors, increased reactivity of the sympathetic system, renal insufficiency and disturbance of the renin-aldosterone relationship [11,22].

Family status "living with children" and role "mother of the family" and "father of the family" were significant factors in the univariate analysis. These findings may be explained by the pressures parents feel in their parenting role, which contribute to parental stress. Parenting and child care can be full of challenges and demands, sleepless nights, and financial and relationship burdens [23]. Parental stress is a set of processes that lead to aversive psychological and physiological responses that result from attempts to adapt to the demands of parenting [24]. As a result, this could adversely affect parents' cardiovascular health [25]. However, after adjustment for confounding variables, parenting was not significantly associated with hypertension.

In the present study, overweight, obesity and abdominal obesity were found to be factors associated with hypertension. According to the ESC/ESH, an increase of 1.7 kg/m2 in BMI or 4.5 cm in waist circumference is associated with a 1 mmHg increase in SBP. Weight gain increases the risk of hypertension by 65-75% [2]. This is explained by the activation of the renin-angiotensin and sympathetic nervous systems and the occurrence of obstructive sleep apnea in obesity [26,27]. However, these factors were not retained after statistical adjustment.

We found that sleep duration of six or less hours was significantly associated with hypertension not only in univariate but also in multivariate analysis. Several observations support the association between hypertension and short sleep duration [28]. The pathophysiological mechanism is not well understood. Sleep deprivation studies have suggested the involvement of the sympathetic nervous system and melatonin. Other mechanisms may include hyperactivity of the renin-angiotensin-aldosterone system, pro-inflammatory responses, and endothelial dysfunction [29,30]. It is likely that cultural and behavioral changes in the Toliara population contribute to this result.

Study strengths and limitations

This study has several limitations. First, there is a time lag between the completion of the study and its publication. The study was conducted in 2019, and since then, recommendations for the definition of hypertension have been updated. Second, the number of individuals with hypertension is small compared with other studies. Third, it did not assess the population's knowledge about hypertension and the type of antihypertensive drugs. This is another important study for future work. Finally, in the statistical evaluation of risk factors, logit linearity and multicollinearity could lead to unstable estimates.

However, the study objectives were met. The prevalence and factors associated with hypertension were described. The sample size was representative of the population of Toliara. More generally, this study contributes to the growing body of literature calling for the study of hypertension in the remotest regions of Africa to become global health research. Furthermore, due to the lack of consensus on the definition of abdominal obesity for the Malagasy population, we adopted Asian criteria. This underscores the importance of determining the appropriate threshold for the Malagasy population.

## Conclusions

The prevalence of hypertension was lower than reported in the literature. After statistical adjustment, the associated factors were older age and short sleep duration. The rates of treated and controlled hypertension were very low, similar to results observed in low-income countries. It is important to emphasize the role of public policy in the prevention and control of cardiovascular risk factors in the population. The study also raises a number of issues to be addressed in future studies, including research to clarify the definition of abdominal obesity and recommendations for the management of hypertension in the Black population living in Africa.

## Appendices

Precision in author collaboration

NA Randriamihangy had the initial idea to conduct the study, developed the methodology and then invited authors from different institutions to collaborate. RMF Randrianarisoa, SMR Randrianambininjanahary and F Ralison responded to this invitation and agreed to participate in the study. SMF Randrianambininjanahary carried out the data collection in the city of Toliara and wrote the original draft. RMF Randrianarisoa developed the analysis and interpretation of statistical data and editing. NA Randriamihangy contributed also to data analysis and interpretation, critical revision, visualisation and validation. F Ralison contributed to critical revision, visualisation, validation and supervision of the article.
